# The Ability of Bacteriophages to Reduce Biofilms Produced by *Pseudomonas aeruginosa* Isolated from Corneal Infections

**DOI:** 10.3390/antibiotics14070629

**Published:** 2025-06-20

**Authors:** Kuma Diriba Urgeya, Dinesh Subedi, Naresh Kumar, Mark Willcox

**Affiliations:** 1 School of Optometry and Vision Science, The University of New South Wales (UNSW), Sydney, NSW 2052, Australia; k.diriba@unsw.edu.au; 2 School of Biological Sciences, Monash University, Clayton, VIC 3800, Australia; dinesh.subedi@monash.edu; 3 School of Chemistry, The University of New South Wales (UNSW), Sydney, NSW 2052, Australia; n.kumar@unsw.edu.au

**Keywords:** *P. aeruginosa*, bacteriophage, phage, biofilm, antibiotic resistance, ocular infection, phage efficacy

## Abstract

*Pseudomonas aeruginosa* (*P. aeruginosa*) is a common antibiotic-resistant pathogen, posing significant public health threats worldwide. It is a major cause of ocular infections, mostly linked to contact lens wear. *P. aeruginosa* often produces biofilm during infections, and these are also associated with antibiotic resistance. Bacteriophage (phage) therapy is emerging as a promising approach for treating multidrug-resistant *P. aeruginosa*. **Objective**: This study aimed to assess the antibiofilm effects of six phages against *P. aeruginosa* biofilms isolated from patients with corneal infections. **Method**: This study examined *P. aeruginosa* strains for their ability to form biofilms using crystal violet assay. Six *P. aeruginosa* bacteriophages (DiSu1 to DiSu6) were used, which were isolated from sewage water in Melbourne, Australia. Spot tests were used to assess phage sensitivity. The effect of phages against *P. aeruginosa* strains was determined using time–kill assay and efficiency of plating. The ability of phage to inhibit biofilm formation over 24 h or reduce preformed biofilms was also studied and confirmed using confocal laser scanning microscopy with Live/Dead staining. **Result**: After 24 h of incubation, all tested *P. aeruginosa* strains formed moderate to strong biofilms. All *P. aeruginosa* strains were sensitive to at least four of the six phages. The highest level of bacterial growth inhibition in the liquid infection model was observed when phages were applied at a multiplicity of infection (MOI) of 100. Certain bacteria/phage combinations were able to inhibit biofilm formation over 24 h, with the combination of strain PA235 and phage DiSu3 producing the highest inhibition (83%) at a MOI of 100. This was followed by the combinations of PA223/DiSu3 (56%), and PA225/DiSu5 (52%). For the reduction in preformed biofilms, the best combinations were PA235 (90%), PA221 (61%), and PA213 and PA225 (57% each), all with DiSu3 after 3 h. However, exposing the biofilm with phages for over 24 h appeared to promote phage resistance as there was evidence of biofilm growth, with the only combination still showing a significant reduction being PA221/DiSu3 (58%) at MOI of 100. **Conclusions**: This study showed that the effect of phages against *P. aeruginosa* is concentration (MOI) dependent. Phages at higher MOI have the ability to disrupt, inhibit, and reduce *P. aeruginosa* biofilms. However, prolonged exposure of the biofilm with phages appeared to promote phage resistance. To enhance phage efficacy and address this form of resistance, further studies utilizing phage cocktails or a combination of phages and antibiotics is warranted.

## 1. Introduction

*P. aeruginosa* is a Gram-negative bacterial pathogen and one of the most common etiologic agents of infections acquired in healthcare centers [1]. It is responsible for high morbidity and mortality rates, along with longer hospital stays and excessive healthcare expenses [2,3,4,5]. It can lead to a variety of health complications, particularly in individuals with compromised immune systems, including severe wound infections, cystic fibrosis infection, ventilator-related pneumonia, catheter-related urinary tract infections, and contact lens-associated corneal infections [6,7,8]. *P. aeruginosa* is the primary bacterial pathogen that causes infection of the cornea (the clear membrane overlying the pupil and colored iris; the infection is called microbial keratitis (MK)) in contact lens users [9,10].

*P. aeruginosa* is a challenging bacterial pathogen due to its antibiotic resistance [2,3,11]. The mechanism of resistance in *P. aeruginosa* is multifaceted, encompassing intrinsic factors like reduced membrane permeability, the presence of efflux pumps and enzyme inactivation, as well as the acquisition of resistance genes via horizontal gene transfer and mutation [12,13,14]. The emergence and dissemination of antibiotic-resistant strains of *P. aeruginosa* is becoming a public health threat [15]. It exhibits inherent and acquired resistance to a wide range of antibiotics, complicating treatment and necessitating the urgent development of new treatment options [16].

Another crucial resistance mechanism of *P. aeruginosa* to antibiotics is biofilm formation, which enables the bacteria to survive in harsh environments [17]. Biofilms, cells encased in a self-produced polymer matrix, act as barriers that restrict therapeutic agents from reaching bacterial cells, making the bacteria much less susceptible to antibiotics compared to their free-floating cell counterparts [14,18]. The decreased metabolism and growth of *P. aeruginosa* biofilm can limit the effectiveness of antibiotics, potentially leading to recurrent and long-lasting infections. This often necessitates the surgical excision of the infected area, extraction of medical devices, and continuous antimicrobial therapy [19,20,21,22,23]. *P. aeruginosa* MK is a more severe ocular infection compared to other bacterial pathogens [24,25], possibly due to biofilm formation. Contact lenses removed from patients with MK have been shown to have microbial biofilms attached to their surface [26,27]. Furthermore, *P. aeruginosa* biofilms on contact lenses caused MK in a rat model [28].

The management of *P. aeruginosa* keratitis relies on rapid and appropriate antibiotic treatment. If the infecting microbe is not susceptible to the first-choice antibiotic there is an increased risk of requiring surgery to maintain vision (penetrating keratoplasty) [29]. Furthermore, infection severity and outcome are affected by increased minimum inhibitory concentrations (MICs) of bacteria to antibiotics; for example, a higher MIC has been associated with increased time for the cornea to re-epithelialize [30]. A recent outbreak of keratitis in the USA caused by an extensively antibiotic-resistant *P. aeruginosa* strain resulted in very severe outcomes [31,32]. The *P. aeruginosa* was resistant to cefepime, ceftazidime, piperacillin–tazobactam, aztreonam, carbapenems, ceftazidime–avibactam, ceftolozane–tazobactam, fluoroquinolones, polymyxins, amikacin, gentamicin, and tobramycin. The infections resulted in four deaths, the surgical removal of infected eyes in four patients, and vision loss in fourteen others. However, the strain remained susceptible to bacteriophages (phages), presenting a potential new treatment option [33].

Phages are viruses that target and kill specific strains of bacteria [34]. Phages are categorized as lytic or lysogenic based on their ability to lyse host bacteria. Lytic phages replicate using the bacterial cell machinery and destroy the host cell, whereas lysogenic phages integrate into the host genes as prophage [35,36]. Phage therapy is being explored as a substitute for conventional antibiotics in treating several bacterial infections [37,38,39,40]. Previous studies have reported that phage therapy for non-ocular infection led to approximately 75% improvement in clinical patients and a 60–80% reduction in bacterial load [41,42]. The use of phages for biofilm disruption and cell lysis is a promising bacterial biofilm treatment in clinical setting [43]. Phages degrade *P. aeruginosa* biofilm matrix by producing enzymes such as polysaccharide depolymerase and directly infect living cells, leading to cell death [44,45,46,47]. Although phages have been used successfully to treat *P. aeruginosa* keratitis in rabbits [48], horses [49], and mice [50,51], and one person has been successfully treated with phage for *Staphylococcus aureus* keratitis [52], these have been ad hoc uses with no detailed research on the spectrum of activity of phages or their ability to kill *P. aeruginosa* isolated from ocular infections when they have formed biofilms. Therefore, the current study aimed to evaluate whether phages active against different strains of *P. aeruginosa* could kill strains once they had formed biofilms and could prevent biofilm formation.

## 2. Results

### 2.1. P. aeruginosa Biofilm Formation and Phage Sensitivity Test

**Figure 1:**
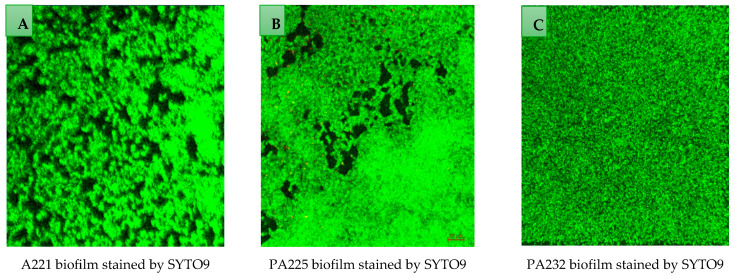
Confocal laser scanning microscopy representative images of 24 h established biofilms of *P. aeruginosa* strains PA221 (image (**A**)), PA225 (image (**B**)) and PA232 (image (**C**)) developed on a glass-bottom fluoro-dish. The preformed biofilms were stained with SYTO 9 which penetrates bacterial cells and stains live bacteria green. In all images (**A**–**C**), the intact matrix of extracellular polymetric substance (EPS) surrounding the bacterial cells is observed.

Twenty-seven strains were screened for biofilm formation and phage sensitivity (Supplementary Table S1). Based on their biofilm formation and phage sensitivity, 10 *P. aeruginosa* strains were included in this study (Table 1). All selected *P. aeruginosa* strains were moderate to strong biofilm producers after 24 h of incubation in the microtiter plate assay. Biofilm formation of *P. aeruginosa* strains was also examined using confocal laser scanning microscopy (Figure 1 shows representative pictures of the biofilms formed by PA221, PA225 and PA232). Among the included *P. aeruginosa* strains, PA216 and PA221 were classified as MDR strains (resistant to an antibiotic in three or more classes, e.g., fluoroquinolone, aminoglycoside, and beta-lactam) [53,54].

**Figure 2:**
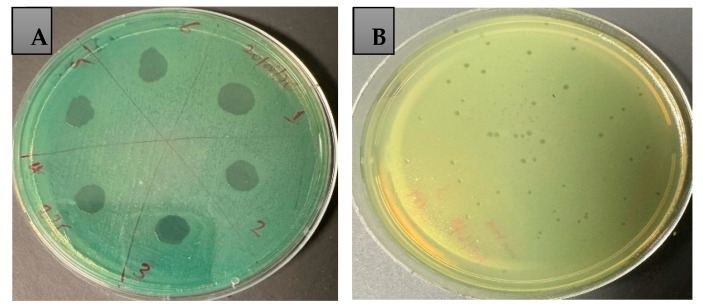
Lytic ability of six different phages against *P. aeruginosa* strain PA235 using spot test method (**A**). Formed plaques on PA235 (DiSu6 titration at dilution of 10^−8^ (**B**)). Plaques formed by all six phages are shown in Supplementary Figure S1.

Based on the spot test assay, all of these strains were sensitive to at least four of the six phages used in this study (Table 1). The spot test assay showed that the phages lysed 67 to 100% of *P. aeruginosa*, displaying a clear zone of inhibition on plates. Six *P. aeruginosa* strains (PA209, PA213, PA221, PA225, PA232, and A235) were sensitive to all six phages, whereas the other strains were resistant to at least one phage (Table 1; Figure 2 and Supplementary Figure S1).

### 2.2. Bacteriophage Propagation and Phage Titration

The spot test revealed that all phages, except for phage DiSu1, could infect *P. aeruginosa* PA01, and produced a wide range of zones of inhibition. DiSu1 was propagated using strain PA235, which was also sensitive to all phages. A large quantity of phage lysates containing highly concentrated phages ranging from 10^7^ plaque-forming units (PFU)/mL to 10^10^ PFU/mL were produced using PA01 and PA235 (Supplementary Figure S1 shows plaque morphology of all phages).

### 2.3. Host Range and Efficiency of Plating (EOP)

Based on phage host range, all ten *P. aeruginosa* strains tested were susceptible to at least four out of six phages evaluated. The lytic activity of phages against these strains ranged from 70% (7/10) to 100% (10/10) (Supplementary Table S3). According to the EOP assay, phage DiSu5 exhibited the highest productivity, showing high EOP values on 67% of strains, followed by phage DiSu3 with 60%, each demonstrating strong activity against six strains. More than 85% of the phages showed medium to high EOP against the tested *P. aeruginosa* strains, indicating efficient lytic activities (Supplementary Table S3). When phages infected the susceptible host strains, the plaques displayed different sizes and morphology on agar plates (Supplementary Figure S1).

### 2.4. Time–Kill Assay

**Figure 3:**
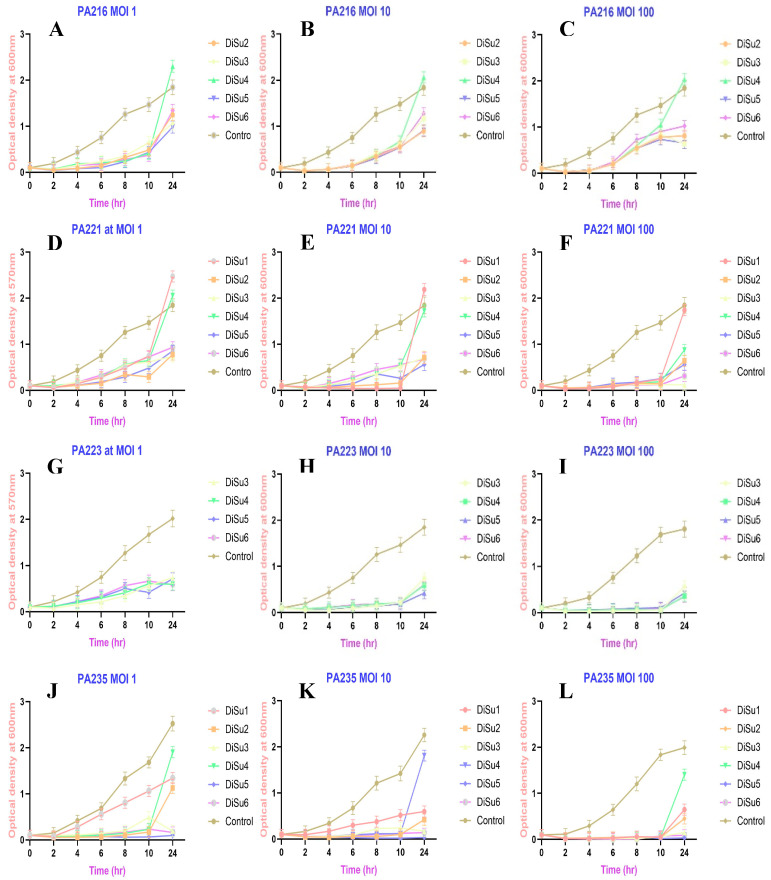
Growth inhibition of *P. aeruginosa* strain PA216 (**A**–**C**), PA221 (**D**–**F**), PA223 (**G**–**I**), and PA235 (**J**–**L**) by individual phages (DiSu1 to DiSu6) in liquid infection model using time–kill assay over 24 h. In each graph, lines of different colors represent the results of *P. aeruginosa* treated with phages and the control (*P. aeruginosa* cells grown in the absence of phages). Phages were applied at MOIs of 1 (10^7^ PFU/mL; **A**,**D**,**G**,**J**), MOIs of 10 (10^8^ PFU/mL; **B**,**E**,**H**,**K**), and MOIs of 100 (10^9^ PFU/mL; **C**,**F**,**I**,**L**). Each data point on the graph represents the average of the three experiments. Error bars show standard deviation of the mean.

The effect of phages against *P. aeruginosa* was concentration and time dependent. The highest reduction in bacterial viability occurred when phages with MOIs of 100 (10^9^ PFU/mL) were used (Figure 3F,I,L), and some *P. aeruginosa* strains showed similar outcomes for phages with different MOIs (Figure 3A–C). All phages significantly inhibited bacterial growth for the first 2 h of coculture, but after that point some bacteria/phage combinations showed growth of the bacteria, especially at lower MOIs (10^7^ PFU/mL). Examples of the growth inhibition are given in Figure 3, with the remainder of the strains shown in Supplementary Figure S2.

The highest inhibition was observed when PA235 was cocultured with DiSu5 at an MOI of 100 (99%), followed by the combination of PA232 + DiSu6 at an MOI of 100 (96%), PA213 + DiSu5 at an MOI of 100 (96%), and PA225 + DiSu5 at an MOI of 100 (95%) (Table 2). Notably, phage DiSu5 demonstrated significant bacterial growth inhibition even at lower MOIs, accounting for 80% and 96% inhibition when combined with PA209 and PA235 at an MOI of 1, respectively (Table 2). The bacterial growth inactivation was also assessed by measuring colony-forming units (CFU)/mL. Phage DiSu5 at an MOI of 100 was the leading inhibitor of PA235 growth. After 24 h of coculture, DiSu5 inactivated bacterial growth from 11.8 log_10_ CFU/mL (phage untreated) to 4.6 log_10_ CFU/mL (phage treated), followed by the combination of PA232 + DiSu6 at MOI 100 (from 11.8 log_10_ to 5 log_10_ CFU/mL), PA213 + DiSu5 (from 11.6 log_10_ to 5.5 log_10_ CFU/mL), PA225 + DiSu5 (from 11.6 log_10_ to 5.8 log_10_ CFU/mL), and PA213 + DiSu3 (from 11.6 log_10_ to 5.7 log_10_ CFU/mL) (Supplementary Figure S3). Phage titers were also assessed after they were cocultured with bacterial strains for 24 h. There was an increase in phage numbers across all MOIs. The highest phage concentrations were observed at MOI 100 when DiSu5 was cocultured with PA235 (from 10^7^ PFU/mL to 8.1 × 10^12^ PFU/mL), at MOI 10 when DiSu5 was cocultured with PA235 (from 10^6^ PFU/mL to 8.7 × 10^9^ PFU/mL), and at MOI 1 when DiSu5 was cocultured with PA209 (from 10^5^ PFU/mL to 7.2 × 10^8^ PFU/mL) (Supplementary Table S4).

### 2.5. Biofilm Inhibition with Phages

**Figure 4:**
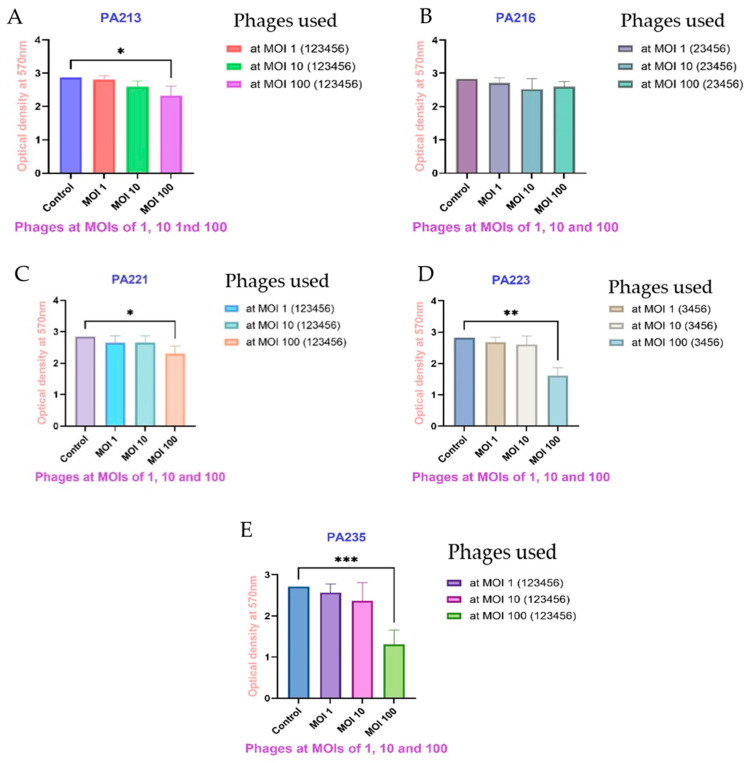
Overview of phages’ ability to inhibit biofilm formation using *P. aeruginosa* strains PA213 (**A**), PA216 (**B**), PA221 (**C**), PA223 (**D**), and PA235 (**E**) when cocultured together for 24 h. The phages used for biofilm inhibition in each *P. aeruginosa* strain are indicated in parentheses beside each graph using the codes 1–6, where 1 = DiSu1, 2 = DiSu2, 3 = DiSu3, 4 = DiSu4, 5 = DiSu5, 6 = DiSu6. Each phage was applied individually in triplicate at each MOI to inhibit biofilm formation and the results are presented as the mean values with 95% confidence intervals. The association between the control and each group was assessed using one-way ANOVA, and statistical significances were indicated as * *p* < 0.05, ** *p* < 0.01, and *** *p* < 0.001. The biofilm inhibition rates of each phage are presented in Table 3.

When different strains of *P. aeruginosa* were cocultured for 24 h with each phage at different MOIs, most of the phages with MOIs of 100 (10^9^ PFU/mL) significantly inhibited *P. aeruginosa* biofilm formation, compared to the control. Figure 4 shows the combined results for selected *P. aeruginosa* against their susceptible phages (Supplementary Figure S4 shows data for other pairings). The results for all pairings are described in detail in Table 3. None of the phages at any MOI (10^7^ PFU/mL, 10^8^ PFU/mL and 10^9^ PFU/mL) could significantly inhibit the biofilm formation of *P. aeruginosa* strains PA216 and PA217. DiSu3 and DiSu5 at MOIs of 100 (10^9^ PFU/mL) were the best biofilm inhibitors against PA235, with reductions of 83% and 74%, respectively (Figure 4, Table 3).

Table 3 shows the inhibition of biofilm formation of all *P. aeruginosa* strains against all phages. After 24 h of biofilm inhibition, the best performing combinations were always with MOI 100 (10^9^ PFU/mL), PA209 + DiSu1, 27% inhibition (*p* = 0.035); PA213 + DiSu3, 27% inhibition (*p* = 0.036); PA216 + DiSu1 17% inhibition, PA217 + DiSu3, 15% inhibition; PA221 + DiSu2, 31% inhibition (*p* = 0.025), PA223 + DiSu3, 56% inhibition (*p* < 0.01); PA225 + DiSu5, 52% inhibition (*p* = 0.02); PA227 + DiSu1, 34% inhibition (*p* = 0.023); PA232 + DiSu6, 36% inhibition (*p* = 0.021); and PA235 + DiSu3, 84% inhibition (*p* < 0.001).

### 2.6. Biofilm Eradication Assay

**Figure 5:**
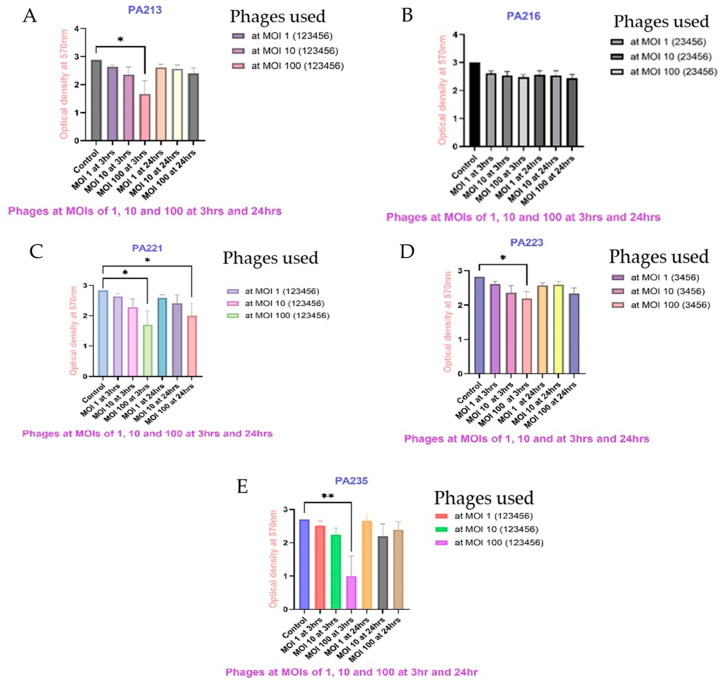
Reduction of 24 h established biofilms formed by *P. aeruginosa* PA213 (**A**), PA216 (**B**), PA221 (**C**), PA223 (**D**), and PA235 (**E**). Each *P. aeruginosa* biofilm was treated with phages at MOIs of 1, 10, and 100 for 3 h and 24 h. The phages used for biofilm reduction in each *P. aeruginosa* strain are indicated beside each graph using the codes 1–6, where 1 = DiSu1, 2 = DiSu2, 3 = DiSu3, 4 = DiSu4, 5 = DiSu5, 6 = DiSu6. Each phage was applied individually in triplicate to remove preformed *P. aeruginosa* biofilms and the results are presented as the mean values with 95% confidence intervals. Biomass reduction was compared with the control (untreated *P. aeruginosa* strains) and statistical significances were assessed using one-way ANOVA, indicated as * *p* < 0.05, ** *p* < 0.01. The biofilm reduction rates of each phage are presented in Table 4.

Preformed *P. aeruginosa* biofilms were exposed to different phages with various MOIs for 3 h and 24 h. Compared to the control, most of the phages significantly reduced preformed *P. aeruginosa* biofilms at MOIs of 100 (10^9^ PFU/mL, *p* < 0.05) after 3 h incubation. Particularly, after 3 h of exposure, phages DiSu1 and DiSu3 removed 88% and 90% of the preformed *P. aeruginosa* PA235 biofilm, respectively (*p* < 0.001) (Figure 5, Table 4). On the other hand, all phages at lower concentrations (MOIs 1, 10^7^ PFU/mL) removed an insignificant percentage of preformed *P. aeruginosa* biofilm (*p* > 0.05; Figure 5, Supplementary Figure S5, and Table 4). As was the case for the ability of phages to prevent formation of biofilms, no phages were able to reduce preformed biofilms of PA216 (Figure 5, Table 4) and PA217 (Supplementary Figure S5, Table 4) after 3 and 24 h exposure. When the time of exposure of preformed *P. aeruginosa* biofilm to phages extended from 3 h to 24 h, some of the phages with higher concentrations still significantly removed preformed *P. aeruginosa* biofilms, while most of the *P. aeruginosa* biofilms became resistant after this longer exposure time to phages (Figure 5, Supplementary Figure S5).

Table 4 shows the inhibition of preformed biofilm by all phages against all strains of *P. aeruginosa* after 3 h and 24 h of exposure. The best performing combinations were always at an MOI of 100, and were between PA209 + DiSu3, MOI = 100 (42% reduction at 3 h, *p* = 0.024); PA213 + DiSu3, MOI = 100 (57% reduction at 3 h, *p* = 0.013); PA216 + DiSu6, MOI = 100 (19% reduction at 24 h); PA217 + DiSu4, MOI = 100 (16% reduction at 24 h); PA221 + DiSu3, MOI = 100 (61% reduction at 3 h, *p* = 0.012); PA221 + DiSu3, MOI = 100 (58% reduction at 24 h, *p* = 0.013); PA223 + DiSu5, MOI = 100 (26% reduction at 3 h, *p* = 0.043); PA225 + DiSu3, MOI = 100 (57% reduction at 3 h, *p* = 0.013); PA227 + DiSu4, MOI = 100 (46% reduction at 3 h, *p* = 0.023); PA232 + DiSu2, MOI = 100 (40% reduction at 3 h, *p* = 0.024); and PA235 + DiSu3, MOI = 100 (90% reduction at 3 h, *p* < 0.01).

### 2.7. Examination of Biofilms Using Confocal Scanning Laser Microscope

**Figure 6:**
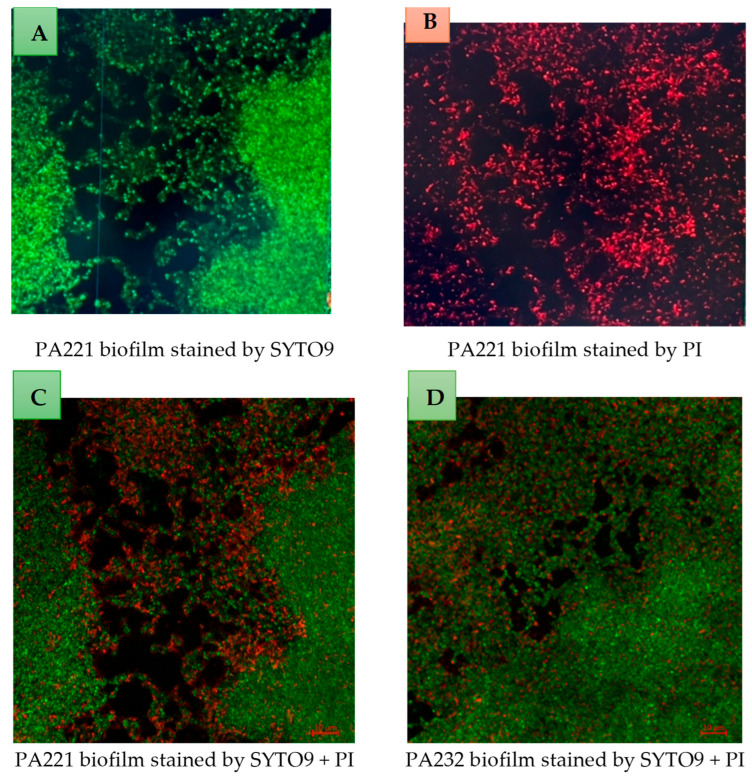
Laser scanning confocal microscopy (CSLM) images of PA221 (**A**–**C**) and PA232 (**D**). Each *P. aeruginosa* strain was adjusted to 10^7^ CFU/mL and incubated on a glass-bottom fluoro-dish for 24 h at 37 °C. The established biofilms were treated with DiSu3 at MOI 100 for 3 h. Images were captured using a 63X objective after staining with SYTO9 and PI for 30 min. Scale bar: 10 µm. Green staining indicates viable bacterial cells, while red indicates dead bacterial cells. DiSu3 significantly killed bacterial cells in the thinner regions of PA221 biofilm (**C**). Image (**C**) split into live bacterial cells (**A**) and dead bacterial cells (**B**). DiSu3 also killed a significant number of bacterial cells in a thinner region of PA232 biofilm (**D**). In all treated biofilms (**C**,**D**), the thick, intact EPS matrix surrounding the bacterial cells protect the bacteria from phage-induced killing within the biofilm.

Phage DiSu3 significantly killed bacterial cells in some areas of the *P. aeruginosa* strain PA221 biofilm. However, areas with a thicker biofilm matrix remained resistant to the phage (Figure 6C). Phage DiSu3 significantly killed bacterial cells in the thinner parts of *P. aeruginosa* strain PA232 biofilm, while the areas with a thicker biofilm matrix remained resistant to the phage (image 6D). In Figure 6C, D the intact EPS matrix surrounding the bacterial cells protect bacteria from phage-induced killing within the biofilm.

## 3. Discussion

Phage therapy is becoming a leading option for treating *P. aeruginosa* due to its specificity, auto-dosing capabilities, and its ability to degrade biofilms [55,56,57,58,59]. The assessment of phage host range is an important prerequisite before considering phages for therapeutic use, and it is typically evaluated using a spot test. In this study all bacterial hosts were lysed by at least four of the six phages, producing clear zones of lysis. This study also evaluated each phage productivity using EOP. More than 85% of phages tested exhibited medium to high EOP values, forming viable plaques. In this study, ten *P. aeruginosa* strains that had previously been isolated from corneal infections were able to produce biofilms, and were sensitive to various phages when they were in suspension. On occasion, phages appeared to stimulate the growth of certain *P. aeruginosa* strains in suspension. For example, phage DiSu4 stimulated the growth of strains PA209, PA213, and PA216 by ≥25% (Table 1). This appears to have some similarities to a finding with *Escherichia coli* and the T7 bacteriophage, where in confined environments, there was growth of *E. coli* [60]. The current study did not find evidence for the stimulation of biofilm formation by the phage, although this can happen in some circumstances [61]. When subcultured together so that the *P. aeruginosa* strains could form biofilms, the phages that were most active were generally not those that were most active when the bacteria were in suspension. For example, for PA209, phage DiSu6 was able to prevent 94% growth when the bacterial cells were in suspension, but only produced a 5% reduction in biofilm formation. Indeed, the only phages that were most effective on bacterial cells in suspension and during biofilm formation were DiSu5 with PA225 and DiSu6 with PA232. Similarly, those phages that were able to reduce preformed biofilms of bacteria after 3 h incubation were also not the same phages that reduced bacterial numbers in suspension, or necessarily, the phages that prevented biofilm formation. All bacterial strains, with the exception of PA221, developed resistance to phages if the phages were incubated with preformed biofilms for 24 h.

Based on the time–kill curves, nearly all strains of *P. aeruginosa* exhibited rapid regrowth within 2 h of coculturing with phages at lower concentrations, while some of them regrew after 10 h when exposed to phages at higher concentrations. In this study, phage DiSu5 and DiSu6, each at a MOI of 100, were the leading inhibitors of PA235 and PA232 growth, respectively. After 24 h of coculture, DiSu5 reduced growth from 11.8 log_10_ to 4.6 log_10_ and DiSu3 reduced growth from 11.5 log_10_ to 5 log_10_ (Supplementary Figure S3). There were increases in phage numbers across all MOIs (Supplementary Table S4). There was no incidence of phage titers decreasing over the 24 h. In general, at an MOI of 1 (10^5^ PFU/mL), all phages increased in number to >10^6^ PFU/mL. When this occurred with a minimum effect (<45%) or no effect on bacterial growth, this implied delayed killing, incomplete lysis or the development of partial resistance. This occurred with the following combinations: DiSu1 plus PA209, PA213, PA221, PA225, PA227 and PA232; DiSu2 plus PA216, PA217, PA225, PA227; DiSu3 plus PA216, PA217, PA225, PA227, DiSu4 plus PA209, PA213, PA217, PA221, PA225, PA227, PA232, and PA235; DiSu5 plus PA217, PA225; DiSu6 plus PA216, PA225. In some cases, this occurred with higher bacterial growth inhibition (>45%), demonstrating effective infection and the greater amplification of phage in presence of bacteria. This occurred for the combinations of DiSu1 plus PS235; DiSu2 plus PA235; DiSu6 plus PA209, PA213, PA221, PA232. In the case of DiSu2 plus PA232, DiSu4 plus PA216, and DiSu5 plus PA216 there was a ≥2 log_10_ increase in phage numbers but <47% bacterial growth inhibition, implying the development of at least partial resistance. Most of the remaining combinations showed ≥50% growth inhibition with a ≥2 log_10_ increase in phage numbers from the effective infection and amplification of phages in the bacteria. The growth of bacteria in the presence of phages may be due to the development of resistance to phages, perhaps as the result of mutation of the phage binding receptors, the masking of the phage receptors, or the activation of restriction-modification systems [62,63,64,65]. This finding aligns with previous studies reporting the rapid emergence of resistant mutants when *P. aeruginosa* is cocultured with phages [66,67,68]. Phage cocktails (i.e., a mixture of different phage types) are a possible strategy to overcome the emergence of phage resistance [69,70]. These effects were also seen with increases in MOI to 10 and 100. *P. aeruginosa* strains PA217 and PA227 usually had a ≤45% reduction in bacterial growth with minimal increases in phage numbers from the initial MOI, implying that the phages may have lost viability, adsorbed debris, or not infected effectively, or the possibility of phage degradation systems and emergence of resistance in these strains. The numbers of phages were only assessed at the end of 24 h growth to evaluate whether phage viability was maintained and to infer amplification during the bacterial killing phase. However, continuous monitoring could provide more detailed dynamics, and this should be conducted in future experiments.

*P. aeruginosa* goes through several processes to produce biofilms. Initial attachment involves flagella, type IV pili, fimbriae, extracellular DNA, and the PsI polysaccharide [71]. It can then decrease the production of some surface moieties such as flagella and type IV and increase the production of extracellular DNA and PsI to form larger biofilms. Many of the processes are under the control of quorum sensing and other regulatory pathways [71]. Some common phage receptors of *P. aeruginosa* are lipopolysaccharide (LPS), flagella, and type IV pili (T4P), as well as efflux pumps [72]. Whilst the current study was not designed to identify the phage receptors of the *P. aeruginosa*, it is possible that a change in the phage susceptibility profile during or after biofilm formation could be the result of changes to LPS and T4P. LPS and flagella synthesis can be decreased during biofilm formation [73,74]. Not only can receptor synthesis decrease, but receptors may also be masked by extracellular polysaccharides (including DNA) produced during biofilm formation. The current study produced some evidence to support this hypothesis, with the confocal microscopy images showing the death of *P. aeruginosa* cells after the addition of phages was limited to thinner, peripheral parts of the biofilms. Although some phages can destroy the extracellular polymeric substances (EPS) in biofilms [75], this development of EPS during biofilm formation may also be the reason for resistance developing over the 24 h of incubation of phages with preformed biofilms.

In the current study, only phages at higher concentrations (10^9^ PFU/mL) effectively inhibited the majority of the *P. aeruginosa* biofilm formation or reduced preformed *P. aeruginosa* biofilms. At a concentration of 10^9^ PFU/mL, phages DiSu3 and DiSu5 were the most effective in inhibiting PA235 biofilm formation, accounting for 84% and 74% inhibition, respectively (Table 3), while phages DiSu1 and DiSu3 showed the highest efficacy in removing preformed PA235 biofilms at the same concentrations, with removal rates of 88% and 90%, respectively (Table 4). Various previous studies have reported that phages effectively inhibited *P. aeruginosa* biofilm formation at different concentrations [36,44,76]. High concentration phage treatment may serve as a promising alternative therapy for preventing *P. aeruginosa* biofilm formation. Similarly, previous studies reported that higher concentrations of phages significantly disrupted preformed *P. aeruginosa* biofilms [77,78,79]. This may be due to the fact that at a higher ratio of the phages to the bacteria, there is an increased interaction between the phages and the bacteria. This is supported by a previous study conducted elsewhere [80]. Several studies have also reported that longer exposure to phages can lead to rapid bacterial regrowth and enhanced biofilm formation [66,67,81]. In contrast, other studies have found that phages effectively suppressed *P. aeruginosa* biofilm even after prolonged exposure [82,83]. This difference may be attributed to the fact that most of these studies used phage cocktails rather than individual phages. This bacterial biofilm resistance may be due to the limited ability of phages to penetrate the intact biofilm matrix [84,85] or due to the EPS matrix affecting the speed of phage movement or blocking their access to the biofilm interior [86]. In this study, phages effectively killed bacterial cells in certain regions of the *P. aeruginosa* biofilm, as observed under a confocal laser scanning microscope. However, areas with a denser biofilm matrix remained resistant to phage penetration (Figure 6).

At this stage, genome sequencing or the further molecular characterization of phages used in this study has not been conducted. Whilst the phage identification, based on plaque morphology and host ranges, indicated each phage was a distinct phage, molecular characterization would definitively identify and classify the phages.

In conclusion, 80% of *P. aeruginosa* included in this study exhibited strong biofilm-forming characteristics. The spot test assay demonstrated that all *P. aeruginosa* were susceptible to four of the six phages tested. This study highlights the concentration-dependent nature of phage activity against *P. aeruginosa* and the importance of exposure time. Phages at higher concentrations were capable of disrupting, inhibiting, and reducing *P. aeruginosa* biofilms, suggesting their potential as a viable substitute for antibiotics in battling bacterial ocular infections. The prolonged exposure of bacteria to phages led to the emergence of phage-resistant bacterial strains. Confocal laser scanning microscopy images revealed that most phages primarily targeted bacterial cells in the thinner biofilm regions, while thicker areas remained resistant. To enhance phage efficacy and overcome this form of resistance, further studies utilizing phage cocktails or a combination of phages and antibiotics are warranted. Furthermore, future studies should examine the reasons why biofilms showed increased resistance to phages, and the change in phage susceptibility from bacterial cells in suspension to those in biofilms.

## 4. Materials and Methods

### 4.1. Assessment of P. aeruginosa Biofilm Formation

With some modifications, the biofilm-forming capabilities of previously isolated *P. aeruginosa* strains were assessed as in a previous study [87]. Briefly, *P. aeruginosa* strains were grown in tryptone soya broth (TSB) (Becton Dickinson, Sparks, MD, USA) with 1% (*w/v*) glucose overnight at 37 °C, then adjusted to 10^6^ colony-forming unit (CFU)/mL by diluting in fresh TSB. Subsequently, 200 µL aliquots of the suspension were added to wells in a sterile 96-well flat-bottom microtiter plate (Corning Life Science, Wu Jang, China) and incubated at 37 °C for 24 h. Non-adherent bacterial cells were discarded, and the wells were gently washed thrice with PBS (NaCl 8 g^−1^, KCl 0.2 g^−1^, Na_2_HPO_4_ 1.15 g^−1^, and KH_2_PO_4_ 0.2 g^−1^). After air drying for 30 min, the biofilms were fixed with absolute methanol and then stained with 0.1% crystal violet (CV) (1% *w/v* Sigma Aldrich, St. Louis, MO, USA) for 15 min. After removing unbound CV by washing with PBS, 200 µL of absolute ethanol was added to the wells, and the solubilized CV was then measured using spectrophotometry at a wavelength of 570 nm. The experiment was performed in triplicate separately for each strain, and the average value was calculated. The cut-off value OD (ODc) for biofilm formation was calculated by adding the arithmetic mean of the negative controls and the triple value of its standard deviation. Sterile TSB was used as a negative control, while *P. aeruginosa* ATCC 19660 was used as a positive control. Biofilm was classified into three categories (weak, moderate, and strong) [87,88].

Strains were also examined for biofilm formation using confocal laser scanning microscopy (Zeiss-LSM-880, Olympus, Tokyo, Japan). *P. aeruginosa* strains were adjusted to 10^7^ CFU/mL, 600 µL of TSB medium was added, and the bacterial suspension was added to a glass-bottom fluoro-dish (poly-L-lysine coated, World Precision Instruments, Ningbo, China) and incubated at 37 °C for 2 h without shaking for biofilm initiation. Then, the fluoro-dish was gently washed two times with PBS to remove non-adherent bacterial cells. Subsequently, 1 mL of sterile TSB was added and incubated for 24 h at 37 °C. Following 24 h of incubation, the fluoro-dish was washed with PBS and bacterial biofilm was stained for 30 min with LIVE/DEAD backlight bacterial viability kit as per the manufacturer’s instructions (Invitrogen, Eugene, OR, USA) and examined using 63x objective confocal laser scanning microscopy.

### 4.2. Bacteriophage Isolation

Phages were isolated from environmental sewage sources at Monash University, Australia. Briefly, 100 mL of sewage water from various sources was mixed in a flask followed by addition of 10 mL of 10× Lysogeny broth (LB; Becton Dickinson, Sydney, Australia) supplemented with 1 mM of both CaCl_2_ and MgCl_2_. Then, overnight-cultured host strains were added into the mixture and incubated overnight at 37 °C. The resulting lysates were purified through centrifugation, filtration, and chloroform treatment [89]. The spot test method was used to determine phage activities based on the production of clear zones of inhibition. The phage lysates were serially diluted using LB and mixed with host strain. A double layer agar method was used to determine the formation of clear plaques after overnight incubation. Then, each plaque on plates were separately picked and resuspended in PBS. After intense vortexing and repeated amplification, the supernatants were collected and the phages titers were determined in a plaque-forming unit (PFU)/mL) using the whole plate titer method and stored at 4 °C for further use.

### 4.3. Bacteriophage Propagation Using Reference Bacteria

Bacteriophages were propagated in accordance with the phage-on tap (PoT) protocol with some modifications [89]. In brief, 200 µL of overnight-cultured *P. aeruginosa* reference strains PA01 or PA235 at an OD of 0.2 was added into LB, supplemented with 1 mM of both calcium chloride and magnesium chloride and incubated for one hour at 37 °C with shaking. Then, 100 µL of phage lysates (~10^8^ PFU/mL) was added to the mixture and further incubated for 5 h at 37 °C with shaking at 120 rpm. After centrifugation at 4000× *g* for 20 min, the supernatants were collected and filtered through a 0.22 mm filter and then mixed with 0.1 volume of chloroform and incubated at room temperature for 10 min. Subsequently, the mixture was centrifuged at 4000× *g* for 5 min. Lastly, the supernatant was collected and the phage titer was determined using the double layer agar method [89] and stored at 4 °C for further use.

### 4.4. Determination of Host Range and Efficiency of Plating (EOP)

The host range was determined by applying a concentrated phage aliquot (~1 × 10^8^ PFU/mL) to actively growing *P. aeruginosa*. In short, 50 µL of an overnight bacterial culture (~1 × 10^8^ CFU/mL) was blended with 5 mL of soft agar (prepared from 1% of TSA, sterilized and cooled to 45–50 °C to prevent solidification) and spread out on plates with TSA. Then, 10 µL of the phage aliquots (10^8^ PFU/mL) were spotted on the soft agar surfaces and kept at 37 °C for 24 h, and the phage host range was determined based on the production of clear zones of inhibition [90,91]. The effectiveness of the phage plating (EOP) was determined using the double-layer agar method. In brief, serially diluted phages were combined with bacterial culture (1 × 10^7^ CFU/mL) in soft agar and incubated at 37 °C for 24 h. Then, the PFU were counted and the EOP was calculated by comparing the phage titers on the target bacteria to those on the reference bacteria. Then, the phages were classified as high productivity if the calculated ratios were >0.5, medium productivity if they were between 0.1 and 0.5, low productivity if they were between 0.001 and 0.1, and inefficient if they were <0.001, as described in a previous study [90].

### 4.5. P. aeruginosa Growth Inhibition in Liquid Medium

The *P. aeruginosa* strains and phages at MOIs of 100, 10, and 1 were cocultured in 96-well microtiter plates. The concentration of *P. aeruginosa* strains was adjusted to 10^7^ CFU/mL (approximately equal to OD of 0.1) and the concentration of each bacteriophage to 10^7^ PFU/mL, 10^8^ PFU/mL, and 10^9^ PFU/mL to give each of the three MOIs, respectively. Then, 100 µL of each *P. aeruginosa* strains and 100 µL of each phage were added to 96-well microtiter plates and incubated at 37 °C with shaking at 100 rpm for 24 h. The growth of *P. aeruginosa* strains was monitored by recording the OD_600nm_ at 2 h intervals for 10 h and then after 24 h of growth (0, 2, 4, 6, 8, 10, and 24 h). A time–kill curve was then constructed. Controls of *P. aeruginosa* growth in the absence of phages and sterile TSB were also performed. Bacterial growth inactivation was assessed by measuring CFU/mL. Initially, 100 µL of pre-adjusted *P. aeruginosa* at a concentration of 10^5^ CFU/mL was added to a 96 microtiter plate, followed by 100 µL of phage solutions at concentration of 10^5^, 10^6^, and 10^7^ PFU/mL to maintain the MOIs of 1, 10, and 100, respectively. Each test was prepared in six replicates. The plate was then incubated at 37 °C with shaking and samples were collected at 2, 4, 6, 8, 10, and 24 h. At each time point, a 100 µL aliquot was removed for CFU quantification. Samples were centrifuged at 10,000 × *g* for 5 min to pellet bacteria; the supernatant was discarded, and the pellet was resuspended in PBS. Serial dilutions were performed in PBS, and 100 µL of the appropriate dilutions was spread onto tryptone soya agar plates and incubated overnight at 37 °C. Finally, colonies were counted and CFU/mL values were plotted against time (hours) to evaluate bacterial viability over time. We also assessed the concentration of the bacteriophages after coculturing with *P. aeruginosa* strains for 24 h. Bacteriophages at initial concentrations of 10^5^ PFU/mL, 10^6^ PFU/mL, and 10^7^ PFU/mL were cocultured with *P. aeruginosa* strains in a 96-well microtiter plate at a concentration of 10^5^ CFU/mL at 37 °C for 24 h with shaking at 150 rpm. After incubation, the cultures were transferred to Eppendorf tubes and centrifuged at 5000× *g* for 10 min. The supernatants were collected, filtered through 0.22 µm filter, serially diluted, and subjected to plaque assay. The results were reported in PFU/mL.

### 4.6. Inhibition of Biofilm Formation by Bacteriophages

The *P. aeruginosa* biofilm inhibition capability of phages was determined following a slight modification of a previously described microtiter plate assay [88,92]. Briefly, 100 µL of refreshed *P. aeruginosa* with a bacterial load of 10^7^ CFU/mL and 100 µL of phage aliquots with various MOI (100, 10 and 1) were added to each well in a 96-well microtiter plate and incubated at 37 °C for 24 h. The contents of the wells were removed and the wells washed once with PBS. Then, the biofilm inhibition activity of the bacteriophages was assessed using the crystal violet assay described above and through measuring the OD at 570 nm. Bacteria alone were used as a positive control for biofilm formation, while TSB alone was used as a negative control. The experiment was conducted in triplicate. The biofilm inhibition percentage was calculated by subtracting the OD_570nm_ of the tested sample (OD_ts_) from the OD_570nm_ of the bacteria control (OD_c_), dividing this result by the OD_c_, and multiplying the result by 100, or (OD_c_ − OD_ts_)/OD_c_ × 100 [92,93].

### 4.7. P. aeruginosa Biofilm Eradication Assay

Aliquots (200 µL) of the bacterial suspensions (10^7^ CFU/mL in TSB) were added to 96-well microtiter plates and incubated at 37 °C for 24 h without agitation to produce biofilms. Then, any planktonic cells were aspirated from the wells and washed three times with PBS. The preformed biofilms were then treated with aliquots of 200 µL of phages at various MOIs (100, 10 and 1). One plate was incubated at 37 °C for 3 h, and the other plate at 37 °C for 24 h. These times were chosen to show effects over a short vs. long time period. Then, after the phage aliquots were removed and washed with PBS, it was fixed with absolute methanol. Bacterial suspension without phages was used as a positive control and sterile TSB as a negative control. The biofilm removal rate at 3 h and 24 h was determined using the CV staining assay. The experiment was conducted in triplicate. The percentage of the removed biofilm was calculated by subtracting the OD_570nm_ of the tested sample (OD_ts_) from the OD_570nm_ of the bacteria control (OD_c_), dividing this result by the OD_c_, and multiplying the result by 100, or (OD_c_ − OD_ts_)/ODc × 100.

The effect of phages was also examined after the formation of biofilm on glass-bottom fluoro-dish plates using confocal microscopy, as described above. After the formation of biofilm for 24 h, the biofilms were treated with 500 µL of phages with MOI of 100, then incubated at 37 °C for 3 h. The fluoro-dish was again washed with PBS and then stained with LIVE/DEAD stain.

### 4.8. Statistical Analysis

Statistical analysis was performed using GraphPad Prism version 10.2.3. Initially, the data fitness for Gaussian distribution was checked using the normality test. One-way ANOVA was used to compare the mean data. A multiple comparison post hoc analysis test was performed with Tukey’s statistical hypothesis testing. The outcome was considered statistically significant if the *p* value was <0.05. All experiments were conducted in triplicate.

## Figures and Tables

**Table 1:** *P. aeruginosa* strains antimicrobial resistance profile, biofilm forming ability, and phage sensitivity test results from spot tests.

*P. aeruginosa Strains*	Antibiotic Resistance [53,54]	Biofilm Formation	DiSu1	DiSu2	DiSu3	DiSu4	DiSu5	DiSu6
PA209	Pip, Imi	Moderate	S	S	S	S	S	S
PA213	Cip, Imi, Cef	Strong	S	S	S	S	S	S
PA216	Cip, Lev, Pip, Imi, Cef, PmB	Strong	R	S	S	S	S	S
PA217	Cip, Lev, Pip, Imi, Cef	Strong	R	S	S	S	S	R
PA221	Cip, Lev, Gen, Tob, Pip, Imi, Cef	Strong	S	S	S	S	S	S
PA223	Cip, Pip, Cef	Strong	R	R	S	S	S	S
PA225	Cip, Lev, Imi	Strong	S	S	S	S	S	S
PA227	Cip, Lev, Imi, Cef	Strong	S	S	S	S	R	R
PA232	ND	Strong	S	S	S	S	S	S
PA235	Cip, Pip, Imi, Cef	Moderate	S	S	S	S	S	S

Strains that were considered to have intermediate resistance to the antibiotics [54] were classified as resistant. Cip = ciprofloxacin, Lev = levofloxacin, Imi = imipenem, Pip = piperacillin, Gen = gentamicin, Tob = tobramycin, Cef = ceftazidime, PmB = polymyxin B. ND = not determined, R = resistant to phages, S = sensitive to phages.

**Table 2:** Percentage of growth inhibition of *P. aeruginosa* strains treated with phages at different MOIs compared to the control (cell growth without phages).

Bacterial Strain		PA209	PA213	PA216	PA217	PA221	PA223	PA225	PA227	PA232	PA235
Phage—MOI = 1	DiSu1	−24.4	−13.9	R	R	−18.7	R	−30.1	−3.0	−16.7	46.8
	DiSu2	68.9	50.7	32.1	18.2	57.8	R	−23.8	−1.4	28.7	55.4
	DiSu3	67.2	61.7	42.6	−22.8	60.0	63.3	−3.0	24.6	69.6	92.3
	DiSu4	−43.8	−86.2	−24.9	20.0	−11.6	70.5	−2.1	−10.6	−1.1	24.5
	DiSu5	79.8	69.4	46.8	−15.1	53.6	64.7	−4.4	R	66.8	96.2
	DiSu6	70.3	57.0	27.2	R	49.3	70.9	−5.8	R	76.3	93.6
Phage—MOI = 10	DiSu1	70.4	30.2	R	R	5.7	R	−9.3	−4.8	−16.7	73.4
	DiSu2	87.2	73.7	51.5	31.2	61.4	R	−12.3	−0.3	28.7	81.3
	DiSu3	79.4	80.3	36.8	−6.5	61.5	59.9	−2.5	71.5	69.6	92.4
	DiSu4	35.6	51.1	−11.9	20.2	6.6	67.7	24.2	0.2	−1.1	19.2
	DiSu5	81.1	80.0	50.6	−6.8	69.7	77.1	23.6	R	66.8	98.4
	DiSu6	82.3	79.7	30.6	R	62.9	67.1	33.4	R	76.3	93.0
Phage—MOI = 100	DiSu1	72.8	58.4	R	R	−34.1	R	−1.5	23.6	−0.6	67.3
	DiSu2	88.7	84.1	55.9	**40.2**	65.2	R	0.0	22.8	75.2	77.2
	DiSu3	92.3	92.4	**65.8**	−4.0	**93.6**	69.1	36.2	**92.5**	94.6	92.4
	DiSu4	56.3	68.2	−10.6	32.5	52.6	80.3	75.7	16.1	39.6	29.0
	DiSu5	89.3	**95.7**	64.6	2.3	69.8	76.6	**95.1**	R	84.8	**98.5**
	DiSu6	**94.2**	89.0	44.6	R	83.1	**81.1**	73.0	R	**96.3**	95.5

**Bold**, indicates the highest amount of growth inhibition for the phage/bacteria combinations. R, indicates that the strain was resistant to infection with that particular phage.

**Table 3:** Biofilm inhibition at various MOIs by coculturing phages with *P. aeruginosa* strains for 24 h compared to the control.

Bacterial Strain	MOI	Percentage of Bacterial Biofilm Inhibition After Coculturing for 24 h					
		DiSu1	DiSu2	DiSu3	DiSu4	DiSu5	DiSu6
PA209	1	1.4	2.5	−4.8	−2.3	−6.0	−2.7
	10	7.5	−0.8	−0.8	−0.8	−0.8	0.6
	100	**27.3**	21.8	21.4	12.6	12.6	5.3
PA213	1	0.0	2.2	10.3	2.9	0.0	0.0
	10	4.9	11.0	11.7	4.9	20.2	6.0
	100	25.1	26.8	**27.4**	7.2	22.0	6.0
PA216	1	R	13.5	5.3	3.3	−1.8	1.7
	10	R	3.1	3.1	14.4	16.3	3.1
	100	R	**16.7**	5.3	10.1	2.5	7.5
PA217	1	R	3.3	6.4	4.9	−3.7	R
	10	R	1.3	8.5	1.3	12.8	R
	100	R	7.0	**15.2**	3.5	8.4	R
PA221	1	−1.3	14.5	17.1	8.1	−1.3	4.9
	10	3.6	3.6	3.6	3.6	22.3	3.6
	100	16.8	**30.6**	26.5	18.4	16.5	6.1
PA223	1	R	R	10.0	−1.9	3.5	10.6
	10	R	R	3.1	3.1	21.8	3.1
	100	R	R	**55.7**	38.4	42.0	35.6
PA225	1	−0.5	6.9	2.4	−1.9	7.3	5.6
	10	3.1	3.1	3.1	3.1	18.7	3.1
	100	18.5	28.4	16.0	16.0	**52.2**	26.7
PA227	1	9.4	3.0	17.7	3.0	R	R
	10	7.7	7.7	7.7	7.7	R	R
	100	**33.7**	14.3	20.0	26.7	R	R
PA232	1	1.5	−1.9	7.5	−1.9	−1.9	−1.9
	10	3.1	3.1	10.0	4.0	3.1	3.1
	100	27.8	6.3	16.0	29.8	25.9	**35.7**
PA235	1	12.6	−0.4	12.4	−6.2	2.6	11.4
	10	−1.1	−1.1	41.3	5.5	9.9	20.6
	100	49.5	41.3	**83.4**	20.6	73.9	32.8

**Bold**, indicates the highest amount of inhibition for the phage/bacteria combinations. R, indicates that the strain was resistant to infection with that particular phage.

**Table 4:** *P. aeruginosa* biofilm reduction rates after treatment with phages at different MOIs for 3 h and 24 h compared to the control (absence of phages).

Bacterial Strain	MOI	Percentage of Bacterial Biofilm Reduction After Exposure to Phages for 3 h						Percentage of Bacterial Biofilm Reduction After Exposure to Phages for 24 h					
		DiSu1	DiSu2	DiSu3	DiSu4	DiSu5	DiSu6	DiSu1	DiSu2	DiSu3	DiSu4	DiSu5	DiSu6
PA209	1	1.9	4.2	8.3	1.9	1.9	1.9	5.4	−2.4	6.6	4.3	8.4	10.7
	10	24.3	20.3	14.0	22.9	15.6	5.4	10.7	13.5	0.3	9.1	9.7	0.3
	100	25.4	25.6	**41.5**	27.7	16.6	13.5	**17.2**	5.5	12.7	11.8	5.5	0.3
PA213	1	7.4	7.4	13.4	7.4	7.4	7.4	11.8	3.3	8.9	6.5	12.0	14.4
	10	19.4	23.5	23.7	7.4	27.3	7.4	12.3	17.8	5.9	11.2	11.3	5.9
	100	27.3	44.6	**56.7**	50.0	54.0	29.3	19.0	15.7	**26.7**	20.4	10.8	7.4
PA216	1	R	8.0	11.9	5.8	5.8	5.8	R	1.6	10.2	8.1	12.0	14.2
	10	R	17.3	5.8	6.1	10.5	12.1	R	16.9	4.2	12.7	13.3	4.2
	100	R	**16.2**	15.0	13.9	9.2	9.2	R	9.2	16.1	15.2	9.2	**18.7**
PA217	1	R	0	8.9	0	0	R	R	−0.3	−0.3	13.7	8.5	R
	10	R	15.8	4.0	4.3	8.9	R	R	6.2	7.1	16.0	11.2	R
	100	R	−0.3	8.6	6.3	**10.4**	R	R	9.6	7.5	**16.3**	14.0	R
PA221	1	6.2	6.2	14.9	6.2	6.2	6.2	11.5	2.1	8.8	10.1	11.0	10.4
	10	24.5	6.2	16.9	29.4	11.5	28.4	15.9	16.5	4.7	29.4	18.6	4.7
	100	60.3	29.1	**61.3**	36.4	25.3	29.4	24.5	18.6	**57.7**	36.4	20.0	20.5
PA223	1	R	R	11.0	5.7	6.5	5.7	R	R	6.6	9.3	12.2	7.7
	10	R	R	16.4	22.4	21.3	6.6	R	R	6.6	9.3	12.2	7.7
	100	R	R	25.2	25.3	**25.6**	12.2	R	R	**21.3**	18.8	18.5	9.1
PA225	1	5.7	11.0	7.5	5.7	5.7	5.7	14.7	1.5	15.5	4.8	8.2	10.7
	10	15.6	19.0	19.4	26.9	13.4	7.5	12.9	5.7	22.1	4.8	8.7	11.8
	100	39.6	22.2	**56.7**	48.6	13.6	22.9	**36.3**	5.7	21.1	18.6	9.1	9.1
PA227	1	10.2	20.0	13.5	10.2	R	R	6.2	6.2	15.5	6.3	R	R
	10	20.3	13.7	26.6	34.2	R	R	15.7	8.8	16.3	23.6	R	R
	100	19.9	34.5	29.8	**45.7**	R	R	13.5	8.8	19.8	**25.5**	R	R
PA232	1	9.5	10.6	5.7	5.7	8.5	5.7	1.5	1.5	6.6	1.5	8.6	5.3
	10	13.2	15.8	13.3	19.2	19.5	5.7	7.9	7.7	20.4	15.2	10.5	7.7
	100	38.2	**40.0**	22.6	25.2	14.7	15.2	7.9	9.1	20.4	**24.8**	9.1	9.1
PA235	1	10.1	13.7	1.6	2.0	6.6	8.2	−2.7	−2.7	−2.7	−2.7	12.0	10.4
	10	14.8	9.4	21.5	23.6	23.8	9.0	44.2	3.9	18.4	14.8	5.3	5.3
	100	88.2	70.2	**90.0**	41.6	44.2	46.4	9.6	12.5	**29.7**	10.1	14.5	16.8

**Bold**, indicates the highest amount of inhibition for the phage/bacteria combinations. R, indicates that the strain was resistant to infection with that particular phage.

## Data Availability

The data presented in this study and in the Supplementary File are available on request to the corresponding author.

## Supplementary Materials

The following supporting information can be downloaded at https://www.mdpi.com/article/10.3390/antibiotics14070629/s1: Figure S1: Plaque production by phages DiSu 1 to 6; Figure S2: Growth inhibition of *P. aeruginosa* strains; Figure S3: Bacterial colony count (CFU/mL) in log_10_ CFU/ml; Figure S4: Biofilm formation inhibition by phages; Figure S5: Biofilm eradication by phages Table S1: Biofilm formation and phage sensitivity of P. aeruginosa strains; Table S2: Phage concentrations in plaque forming units per ml (PFU/mL); Table S3: Phages efficiency of plating (EOP) and host ranges; Table S4: Determination of phage titer after co-culturing with *P. aeruginosa* strains for 24 h.
