# Supplementary material for: The Ability of Bacteriophages to Reduce Biofilms Produced by Pseudomonas aeruginosa Isolated from Corneal Infections
Source: Antibiotics (Basel). 2025 Jun 20;14(7):629. doi: 10.3390/antibiotics14070629 (PMC12291712; doi:10.3390/antibiotics14070629)
Supplement: Supplementary file 1 [file antibiotics-14-00629-s001.zip › antibiotics-3529837-supplementary.pdf]

Supplementary Figure S1. Plaque production by phages DiSu 1 to 6

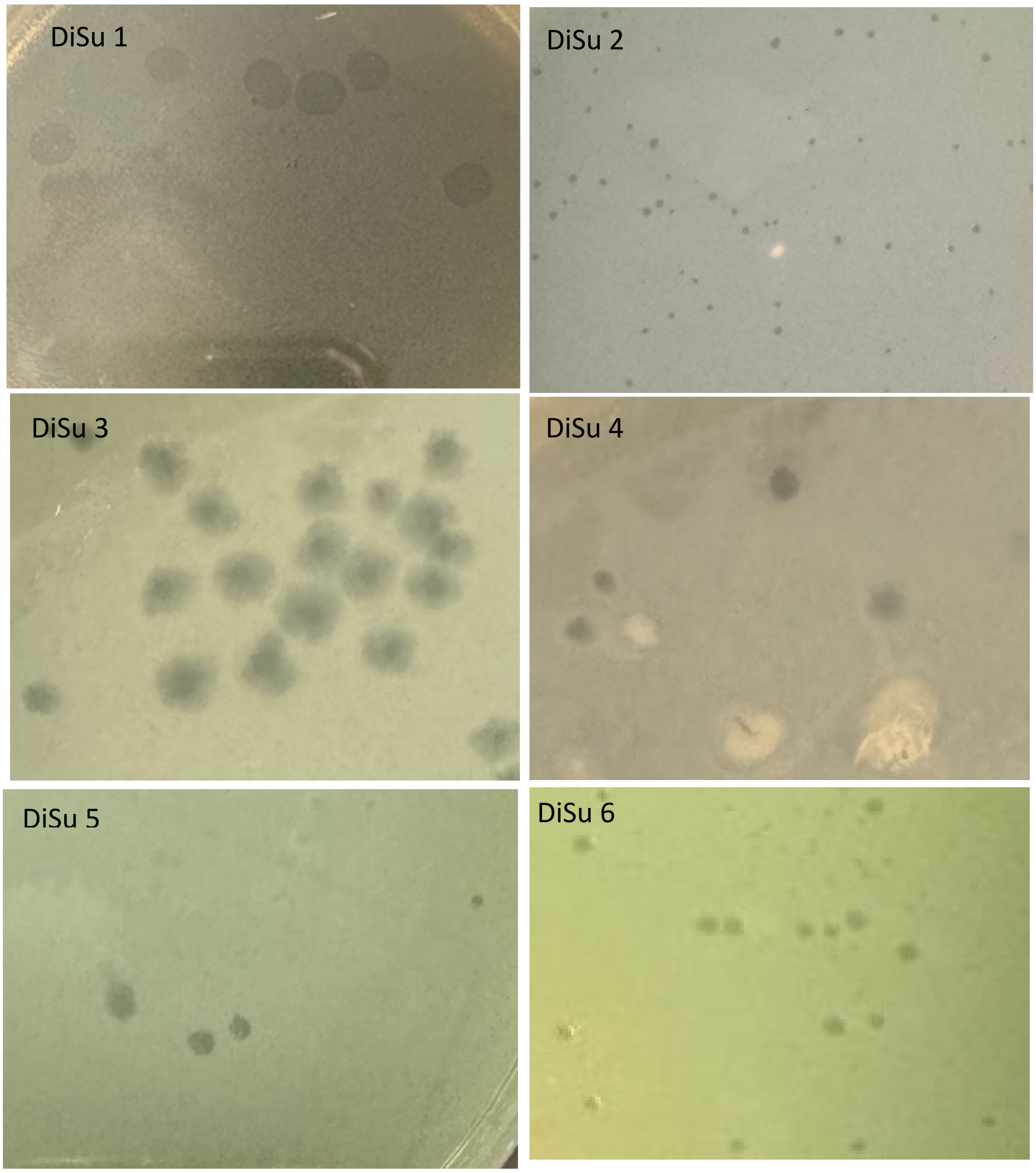

Supplementary Figure S2. Growth inhibition of *P. aeruginosa* strains

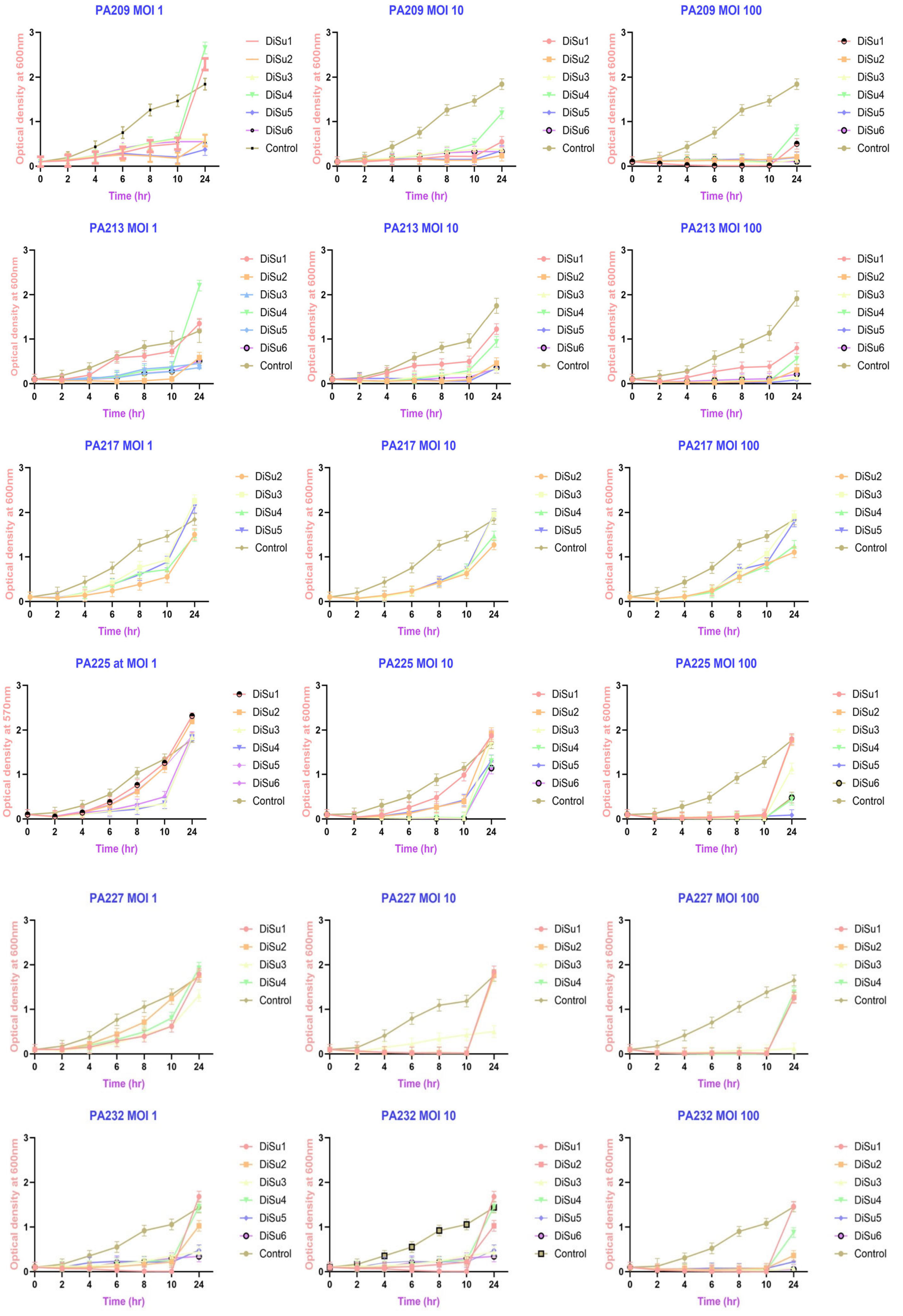

Supplementary Figure S3: Bacterial colony count (CFU/mL) in log_{10} CFU/ml

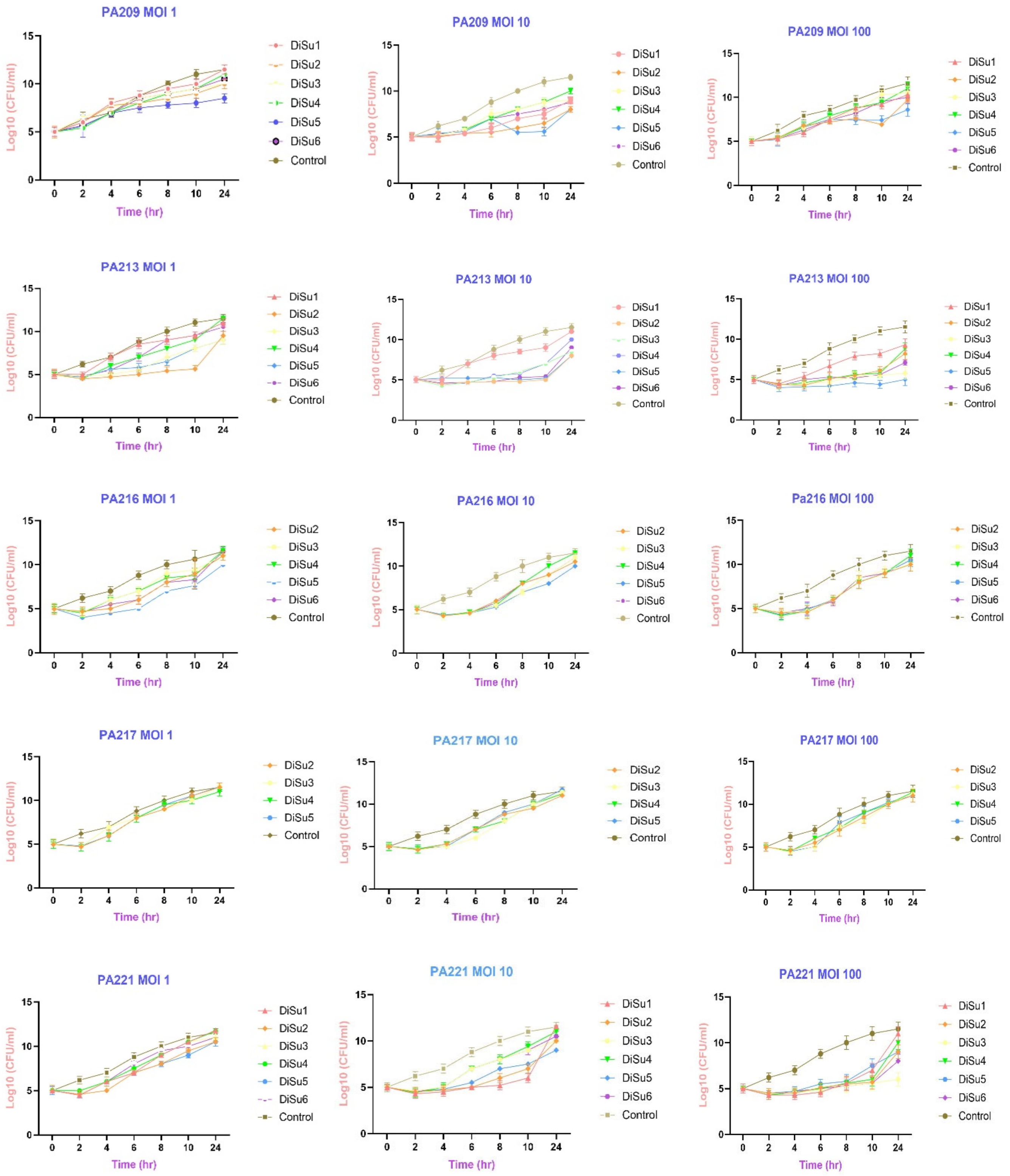

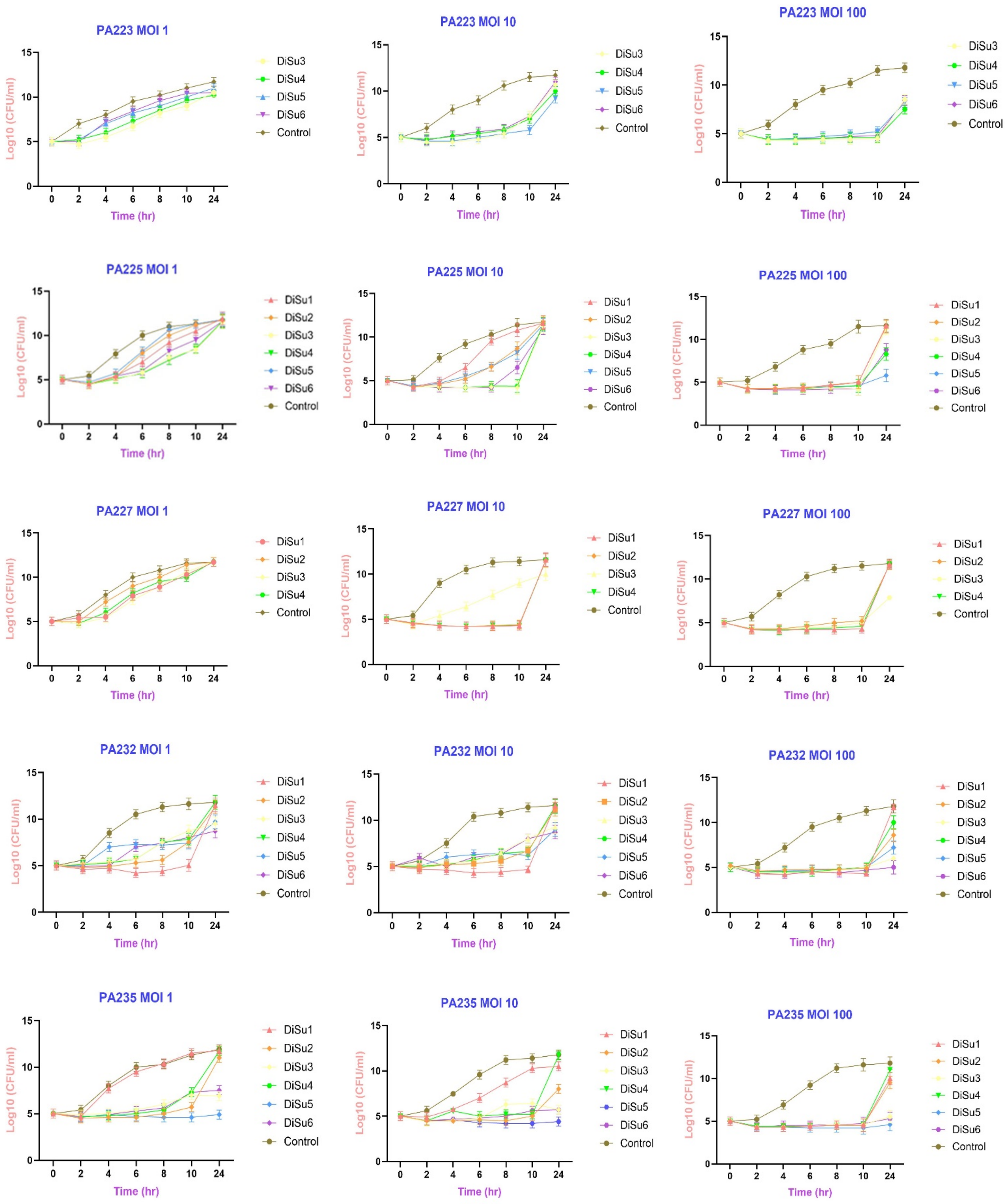
PA223 MOI 1
PA223 MOI 10
PA223 MOI 100
PA225 MOI 1
PA225 MOI 10
PA225 MOI 100
PA227 MOI 1
PA227 MOI 10
PA227 MOI 100
PA232 MOI 1
PA232 MOI 10
PA232 MOI 100
PA235 MOI 1
PA235 MOI 10
PA235 MOI 100
Log10 (CFU/ml)
Time (hr)
DiSu1
DiSu2
DiSu3
DiSu4
DiSu5
DiSu6
Control

Supplementary Figure S4. **Biofilm formation inhibition by phages**

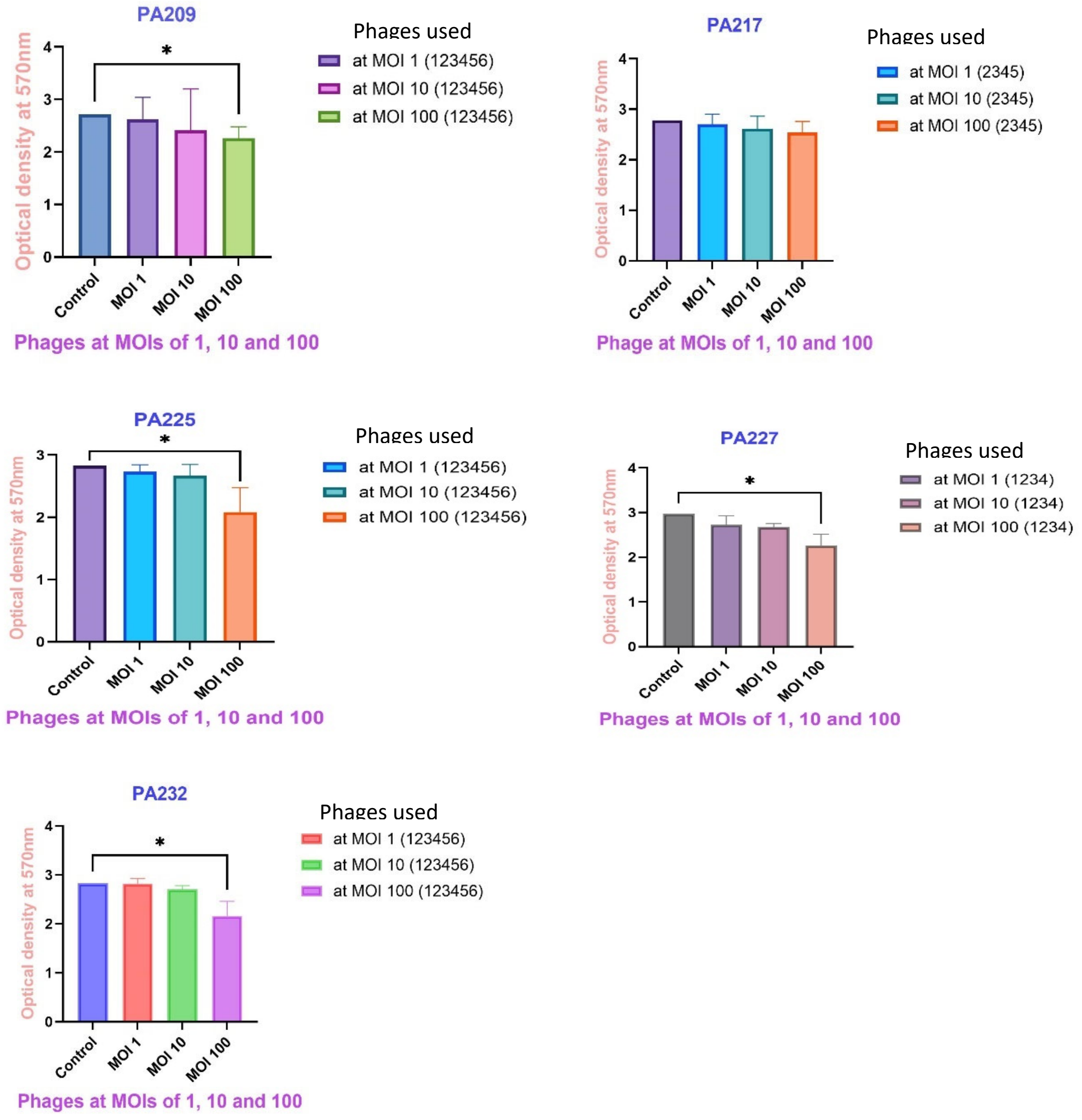

Supplementary Figure S5. **Biofilm eradication by phages**

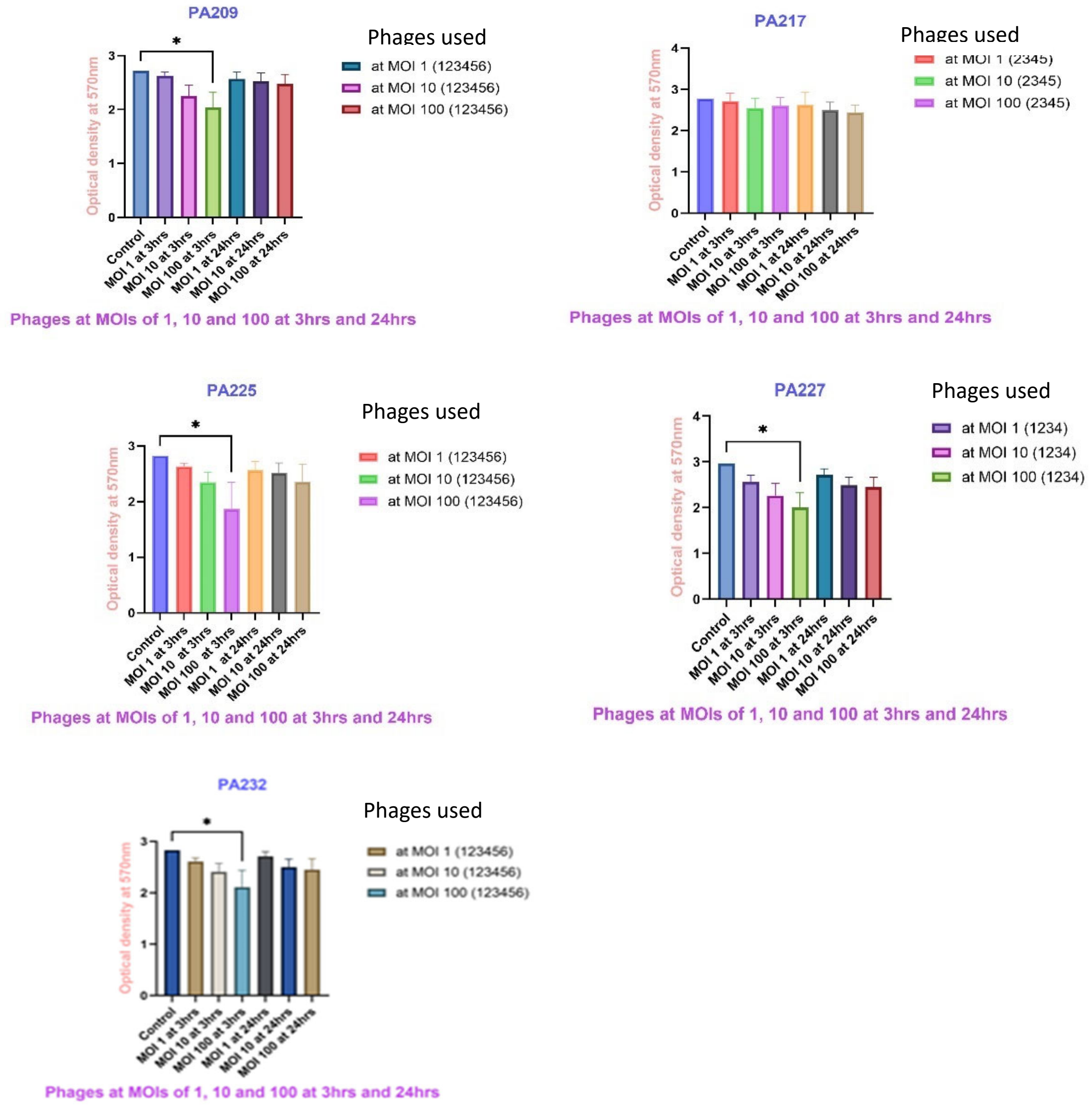

Supplementary Table S1: **Biofilm formation and phage sensitivity of *P. aeruginosa* strains**

P. aeruginosa strain	Biofilm formation	Phage sensitivity test						Number of sensitive phages	Those included for further testing
		1	2	3	4	5	6		
PA165	Strong	R	S	R	R	R	R	1	
PA170	Weak	R	R	R	R	S	S	2	
PA172	Strong	R	S	R	R	R	S	2	
PA174	Strong	R	S	R	R	R	R	1	
PA176	Strong	R	S	R	R	R	R	1	
PA181	Strong	R	S	R	R	R	S	2	
PA208	Strong	R	S	R	R	S	R	2	
PA209	Moderate	S	S	S	S	S	S	6	Selected
PA210	Strong	R	R	S	S	R	R	2	
PA212	Moderate	R	R	R	R	S	R	1	
PA213	Strong	S	S	S	S	S	S	6	Selected
PA216	Strong	R	S	S	S	S	S	5	Selected
PA217	Strong	R	S	S	S	S	R	4	Selected
PA218	Strong	R	R	R	R	R	R	0	
PA219	Strong	S	R	S	S	R	R	3	
PA220	Strong	R	R	S	S	R	R	2	
PA221	Strong	S	S	S	S	S	S	6	Selected
PA223	Strong	R	R	S	S	S	S	4	Selected
PA224	Strong	R	R	R	R	S	S	2	
PA225	Strong	S	S	S	S	S	S	6	Selected
PA227	Strong	S	S	S	S	R	R	4	Selected
PA228	Strong	S	S	R	S	S	S	3	
PA229	Strong	R	S	R	R	S	R	2	
PA231	Strong	R	R	R	R	R	R	0	
PA232	Strong	S	S	S	S	S	S	6	Selected
PA233	Strong	R	R	R	R	S	S	2	
PA235	Moderate	S	S	S	S	S	S	6	Selected

Supplementary Table S2: Phage concentrations in plaque forming units per ml (PFU/ml)

Phage number	Concentration (PFU/ml)
DiSu1	$7x10^{7}$
DiSu2	$9.8x10^{9}$
DiSu3	$5.2x10^{9}$
DiSu4	$2.1x10^{7}$
DiSu5	$2.8x10^{10}$
DiSu6	$4.7x10^{10}$

Supplementary Table S3. Phages efficiency of plating (EOP) and host ranges

	Bacteriophages					
	DiSu1	DiSu2	DiSu3	DiSu4	DiSu5	DiSu6
	Number of *P. aeruginosa* strains lyzed in spot test (maximum = 10)					
	7	9	10	10	9	8
	Number of *P. aeruginosa* strains					
High EOP	3	4	6	5	6	4
Medium EOP	3	4	4	5	3	3
Low EOP	1	1	0	0	0	1
Inefficient EOP	0	0	0	0	0	0

Supplementary Table S4: Determination of phage titer after co-culturing with *P. aeruginosa* strains for 24 hours

Phage	Initial phage concentration	Phage concentration (PFU/mL) after co-culture with different strains of *P. aeruginosa* for 24 hours									
		**PA209**	**PA213**	**PA216**	**PA217**	**PA221**	**PA223**	**PA225**	**PA227**	**PA232**	**PA235**
Initial MOI = 1											
DiSu1	10^{5}	$4.6x10^{6}$	$4.2x10^{6}$	ND	ND	$8.1x10^{6}$	ND	$5.5x10^{6}$	$2.3x10^{6}$	$3.7x10^{6}$	$4.4x10^{6}$
DiSu2	10^{5}	$6.5x10^{7}$	$9.5x10^{7}$	$2.8x10^{6}$	$3x10^{6}$	$3.5x10^{7}$	ND	$4.3x10^{6}$	$3.2x10^{6}$	$4.2x10^{7}$	$4.8x10^{6}$
DiSu3	10^{5}	$7.8x10^{7}$	$8.4x10^{7}$	$8.4x10^{6}$	$2.5x10^{6}$	$3.6x10^{6}$	$6.6x10^{7}$	$2.8x10^{6}$	$4.6x10^{6}$	$7.6x10^{7}$	$7.6x10^{7}$
DiSu4	10^{5}	$5.8x10^{6}$	$4.3x10^{6}$	$3.1x10^{7}$	$3.7x10^{6}$	$4.7x10^{6}$	$5.2x10^{7}$	$4.2x10^{6}$	$3.8x10^{6}$	$4.4x10^{6}$	$5.9x10^{6}$
DiSu5	10^{5}	$7.2x10^{8}$	$6.8x10^{8}$	$4.2x10^{7}$	$3.2x10^{6}$	$5.4x10^{7}$	$4.8x10^{7}$	$6.8x10^{6}$	ND	$7.5x10^{7}$	$5.7x10^{8}$
DiSu6	10^{5}	$8.5x10^{6}$	$4.5x10^{6}$	$6.2x10^{6}$	ND	$4.5x10^{6}$	$5.7x10^{7}$	$4.1x10^{6}$	ND	$9.3x10^{6}$	$6.8x10^{7}$
Initial MOI = 10											
DiSu1	10^{6}	$5.7x10^{6}$	$7.4x10^{6}$	ND	ND	$9.2x10^{6}$	ND	$7.4x10^{6}$	$5.7x10^{6}$	$3.2x10^{7}$	$7.8x10^{7}$
DiSu2	10^{6}	$9.5x10^{7}$	$9.7x10^{7}$	$6.3x10^{6}$	$6.2x10^{6}$	$5.6x10^{7}$	ND	$8.8x10^{6}$	$7.5x10^{6}$	$8.8x10^{7}$	$7.5x10^{7}$
DiSu3	10^{6}	$8.6x10^{7}$	$8.8x10^{7}$	$9.2x10^{6}$	$8.4x10^{6}$	$5.3x10^{7}$	$8.1x10^{7}$	$3.2x10^{7}$	$3.7x10^{7}$	$4.6x10^{8}$	$5.5x10^{9}$
DiSu4	10^{6}	$6.5x10^{6}$	$8.6x10^{6}$	$4.2x10^{7}$	$4.9x10^{6}$	$7.7x10^{6}$	$8.9x10^{7}$	$7.6x10^{6}$	$6.9x10^{6}$	$9.5x10^{6}$	$2.6x10^{7}$
DiSu5	10^{6}	$8.6x10^{8}$	$9.1x10^{8}$	$6.8x10^{7}$	$7.6x10^{6}$	$5.9x10^{8}$	$3.1x10^{8}$	$3.3x10^{7}$	ND	$5.6x10^{8}$	$8.7x10^{9}$
DiSu6	10^{6}	$5.3x10^{7}$	$7.6x10^{6}$	$8.1x10^{6}$	ND	$7.1x10^{6}$	$8.6x10^{7}$	$2.7x10^{7}$	ND	$6.5x10^{7}$	$9.1x10^{7}$
Initial MOI = 100											
DiSu1	10^{7}	$6.4x10^{7}$	$3.1x10^{7}$	ND	ND	$6.9x10^{7}$	ND	$6.7x10^{7}$	$6.1x10^{7}$	$6.7x10^{7}$	$3.4x10^{8}$
DiSu2	10^{7}	$3.2x10^{8}$	$2.7x10^{8}$	$7.1x10^{7}$	$6.8x10^{7}$	$8.3x10^{7}$	ND	$5.9x10^{7}$	$5.6x10^{7}$	$5.9x10^{8}$	$5.5x10^{8}$
DiSu3	10^{7}	$9.5x10^{7}$	$4.3x10^{9}$	$8.8x10^{7}$	$8.5x10^{7}$	$7.8x10^{7}$	$9.2x10^{8}$	$8.7x10^{7}$	$7.4x10^{7}$	$6.7x10^{9}$	$4.9x10^{10}$
DiSu4	10^{7}	$7.1x10^{7}$	$7.6x10^{7}$	$7.9x10^{7}$	$7.7x10^{7}$	$5.9x10^{7}$	$3.4x10^{9}$	$5.8x10^{8}$	$4.9x10^{7}$	$5.8x10^{8}$	$4.1x10^{8}$
DiSu5	10^{7}	$2.7x10^{9}$	$5.9x10^{9}$	$4.2x10^{8}$	$8.2x10^{7}$	$7.8x10^{8}$	$2.9x10^{9}$	$2.9x10^{9}$	ND	$5.9x10^{11}$	$8.1x10^{12}$
DiSu6	10^{7}	$3.0x10^{8}$	$5.2x10^{8}$	$7.3x10^{7}$	ND	$4.9x10^{7}$	$9.3x10^{8}$	$3.3x10^{8}$	ND	$2.8x10^{8}$	$7.3x1^{10}$

ND = not determined as strain is resistant to the phage
